# Structural and Functional Cerebral Correlates of Hypnotic Suggestibility

**DOI:** 10.1371/journal.pone.0093187

**Published:** 2014-03-26

**Authors:** Alexa Huber, Fausta Lui, Davide Duzzi, Giuseppe Pagnoni, Carlo Adolfo Porro

**Affiliations:** Department of Biomedical, Metabolic and Neural Sciences, University of Modena and Reggio Emilia, Modena, Italy; University of Cardiff, United States of America

## Abstract

Little is known about the neural bases of hypnotic suggestibility, a cognitive trait referring to the tendency to respond to hypnotic suggestions. In the present magnetic resonance imaging study, we performed regression analyses to assess hypnotic suggestibility-related differences in local gray matter volume, using voxel-based morphometry, and in waking resting state functional connectivity of 10 resting state networks, in 37 healthy women. Hypnotic suggestibility was positively correlated with gray matter volume in portions of the left superior and medial frontal gyri, roughly overlapping with the supplementary and pre-supplementary motor area, and negatively correlated with gray matter volume in the left superior temporal gyrus and insula. In the functional connectivity analysis, hypnotic suggestibility was positively correlated with functional connectivity between medial posterior areas, including bilateral posterior cingulate cortex and precuneus, and both the lateral visual network and the left fronto-parietal network; a positive correlation was also found with functional connectivity between the executive-control network and a right postcentral/parietal area. In contrast, hypnotic suggestibility was negatively correlated with functional connectivity between the right fronto-parietal network and the right lateral thalamus. These findings demonstrate for the first time a correlation between hypnotic suggestibility, the structural features of specific cortical regions, and the functional connectivity during the normal resting state of brain structures involved in imagery and self-monitoring activity.

## Introduction

Hypnosis has attracted growing interest in cognitive neuroscience [1]. Hypnotic suggestibility (HS) is a stable cognitive trait that refers to the generalised tendency to respond to hypnotic suggestions, i.e. suggestions administered following a hypnotic induction procedure [1] (we use the terminology proposed by Kirsch and Braffman [2]). The HS trait varies considerably among individuals, is in part heritable [3] and can be reliably measured with standardized scales [4]. Behavioural and physiological studies have documented HS-related differences in many cognitive and sensory-motor functions including imagery, attention, and postural control [5], [6], also during normal waking state (i.e., without hypnotic induction) and in the absence of specific suggestions. On these grounds, HS can be hypothesized to be related to specific brain signatures.

Relatively little is known, however, about the neural bases of HS. Studies using functional magnetic resonance imaging (fMRI) and electroencephalography (EEG) have demonstrated that individuals with high HS scores (Highs), but not individuals with low HS scores (Lows), show significant changes in cerebral activation or functional connectivity (FC) in response to hypnotic suggestions for altered perception [7], [8], and when they are in (vs. out of) hypnosis during attentional tasks [9], [10] or during rest [11], [12]. Some of these studies found changes in activity or FC in Highs in the dorsal anterior cingulate cortex (dACC) and in the dorsolateral prefrontal cortex (DLPFC). Hoeft et al. [13] published the only MRI study, at the time of the present writing, exploring differences between Highs and Lows in resting state FC during the normal waking state. They assessed FC of three brain networks involving the ACC and DLPFC and found that Highs, compared to Lows, had stronger resting state FC between the left DLPFC and the dACC. Interestingly, a recent study demonstrated that disruption of left DLPFC activity by means of low-frequency repetitive transcranial magnetic stimulation resulted in increased responses to hypnotic suggestions [14].

These findings are consistent with the hypothesis that Highs, compared to Lows, feature a frontal attention system that can be more flexibly deployed, for example depending on suggestions, and that hypnotic induction may impinge on regions involved in executive processes, subserving selective attention and conflict resolution [9], [14], [15].

However, studies including only Highs and Lows ignore about one half of the population, which falls in the medium range of HS (Mediums) [16].

In the present MRI study, we aimed to investigate more comprehensively the structural and functional cerebral correlates of HS in healthy female volunteers. To this end, we searched for HS-related differences in waking resting state FC in a set of intrinsic brain networks commonly identified in fMRI studies [17], using the individual HS scores as a linear regressor to assess relationships based on the entire, naturally occurring distribution of HS. Specifically, we assessed FC for the ten prototypical brain networks identified by Smith et al. [17] based on resting fMRI data, as well as on a large number of functional activation studies. We expected to find HS-related differences in FC (a) between the left DLPFC and brain networks including the ACC, which would confirm Hoeft et al.'s [13] findings, and (b) in brain areas involved in mental imagery, given the previously reported links between HS and both vivid imagery [18] and fantasy proneness [19]. Furthermore, we explored possible HS-related differences in brain structure, by estimating local gray matter volume (GMV) using voxel-based morphometry (VBM).

## Materials and Methods

### Ethics statement

All experimental procedures were conducted in conformity to the ethical principles of the Declaration of Helsinki and were approved by the committee on ethics of Modena. All subjects gave their written informed consent to participate in the study.

### Subjects and procedures

As studies on HS frequently report gender-related differences, including marginally higher average HS scores in women compared to men [20], we assessed only women in this study. Subjects were recruited via advertisements among students and staff of the Modena University and among blood donors of the Italian Association for Blood Donors (AVIS) in Modena. Thirty-seven healthy women without any history of neurological or psychiatric illness (6 post-menopausal; 3 ambidextrous, 4 left-handed; age range  =  19–60 years, mean age 37.3 years) participated in an MR session followed by a behavioural session on a different day, with up to one month between the two sessions.

During the behavioural session, handedness was assessed with the Edinburgh inventory [21], trait anxiety with the State-Trait Anxiety Inventory Form Y (STAI-Y2) [22], and mental absorption with the Tellegen Absorption Scale (TAS) [23]. HS was assessed by one of the authors (A.H.) with the Italian version of the Stanford Hypnotic Susceptibility Scale – Form A (SHSS:A), which has demonstrated good test-retest reliability after two days (r =  0.83 in a sample including both genders) [24] and acceptable test-retest reliability after 25 years (r =  0.73 in females) [4]. Subjects were not pre-selected for SHSS score. The order of administration of the scales was the same for all subjects – Edinburgh inventory, STAI-Y2, TAS, SHSS:A.

### Voxel-based morphometry

Using a 3T Philips Achieva MR scanner, a high-resolution T1-weighted structural brain image was acquired (repetition time [TR] = 35 ms; flip angle [FA] = 50°; echo time [TE] = 5.7 ms; sense factor = 1.7/2.0/1.7; isotropic voxel size = 0.5 mm; 360 sagittal slices without gap; matrix = 480 × 480 voxels; field-of-view [FOV] = 240 × 240 mm; acquisition time = 9 min).

Gray matter volume (GMV) was assessed by voxel-based morphometry (VBM), using the VBM8 toolbox (http://dbm.neuro.uni-jena.de/vbm8/) implemented in the SPM8 software package (Wellcome Department of Imaging Neuroscience, London, UK), running under MATLAB (R2010b). Briefly, the individual structural images were segmented into gray matter, white matter and cerebro-spinal fluid, spatially normalized to the MNI space using the DARTEL approach [25], with intensity modulation by the amount of contraction to obtain the local GMV corrected for individual brain size, and spatially smoothed using a 8-mm FWHM Gaussian kernel. In the statistical group analysis, the individual (centered) HS levels were used as a linear predictor for the individual differences in local GMV. Age and a measure of the subject's tendency to move during MR scans, estimated from the functional scans, were included as confound regressors in the analysis. A double statistical threshold of voxel-wise p < 0.005 and cluster size ≥ 1098 voxels, as determined by the AFNI routine AlphaSim using 10,000 Monte Carlo simulations (http://afni.nimh.nih.gov/afni/doc/manual/AlphaSim), was chosen to obtain an experiment-wise alpha < 0.05 (corrected for multiple comparisons).

### Functional connectivity

To measure spontaneous blood oxygenation level dependent (BOLD) signal fluctuations at rest, two runs were acquired while subjects lay in the scanner relaxed with their eyes closed (for each run: 200 gradient-echo echo-planar imaging [EPI] functional volumes; TR = 2000 ms; TE = 30 ms; FA = 80°; isotropic voxel size = 3.6 mm; 35 axial slices with interleaved acquisition in the +z direction without gap; acquisition matrix 80 × 63; FOV = 286 × 229 mm; acquisition time = 7 min).

During the entire scanning session, breathing was recorded with a thoracic belt and heart rate was measured with a photo-plethysmometer placed on the index finder of the right hand.

Functional data were preprocessed using the AFNI software package (http://afni.nimh.nih.gov/afni) [26]. For each subject, retrospective image correction (RETROICOR) with regressors constructed on a slice-by-slice basis was applied to reduce physiological noise related to heartbeat and respiration [27]. All functional volumes were then slice-time corrected and realigned to the first acquired volume. Signal noise related to local white matter fluctuation and hardware instabilities was modeled using the ANATICOR procedure [28]. The EPI volumes were finally spatially warped to the Talairach template and smoothed with a 6-mm FWHM Gaussian kernel.

Functional connectivity (FC) was assessed with the FSL dual regression procedure [29], using a publicly available template of 10 representative spatial resting state networks (RSNs), as identified by Smith et al. [17] (http://www.fmrib.ox.ac.uk/analysis/brainmap+rsns/), after a preliminary transformation from MNI to Talairach space. The 10 maps include three visual networks (corresponding to medial occipital cortex, occipital pole, and lateral occipital areas, respectively), the default mode network (DMN), the cerebellum, a sensorimotor and an auditory network, an executive control or saliency network (including medial frontal areas, anterior cingulate and paracingulate cortex, as well as the anterior insular cortex), and two (left and right) fronto-parietal networks.

For each run, the dual regression procedure yielded a subject-specific set of spatial maps corresponding to the projection of the template RSNs onto the subject's EPI data. In the statistical group analysis, the (centered) vector of individual HS levels was used as a linear regressor for these individual maps, averaged voxel-wise over the two runs, for each of the 10 RSNs. This procedure tested for positive and negative correlations between HS and FC.

Finally, to assess whether HS-related structural differences in GMV were accompanied by differences in FC in the same regions, seed-based FC analysis was performed using the AFNI 3dGroupInCorr procedure. The seed signals were obtained by averaging the BOLD signal within a sphere of 6-mm radius around the Talairach coordinates of the peak voxels showing HS-related effects in the VBM analysis. Seed-based FC was expressed as the Fisher-transformed Pearson correlation coefficients between the seed signal and all other voxels of the brain. For all FC analyses, a double statistical threshold (voxel-wise p < 0.01 and cluster size ≥ 78 voxels, as determined by AlphaSim with 10,000 Monte Carlo simulations) was used to obtain an alpha < 0.05 (corrected for multiple comparisons) (see details on the procedure at http://afni.nimh.nih.gov/afni/doc/manual/AlphaSim and in [30]).

## Results

### Behavioural results

The HS score ranged from 0 to 10 (mean 3.8) and its distribution was positively skewed, including 49% Lows (HS <4), 41% Mediums (HS =  4–7) and 11% Highs (HS >7). HS was independent of age, educational level, handedness, trait anxiety, mental absorption, average movement during the functional scans, and global correlation among all voxels (“gcor” index [31]).

### Voxel-based morphometry

**Figure 1:**
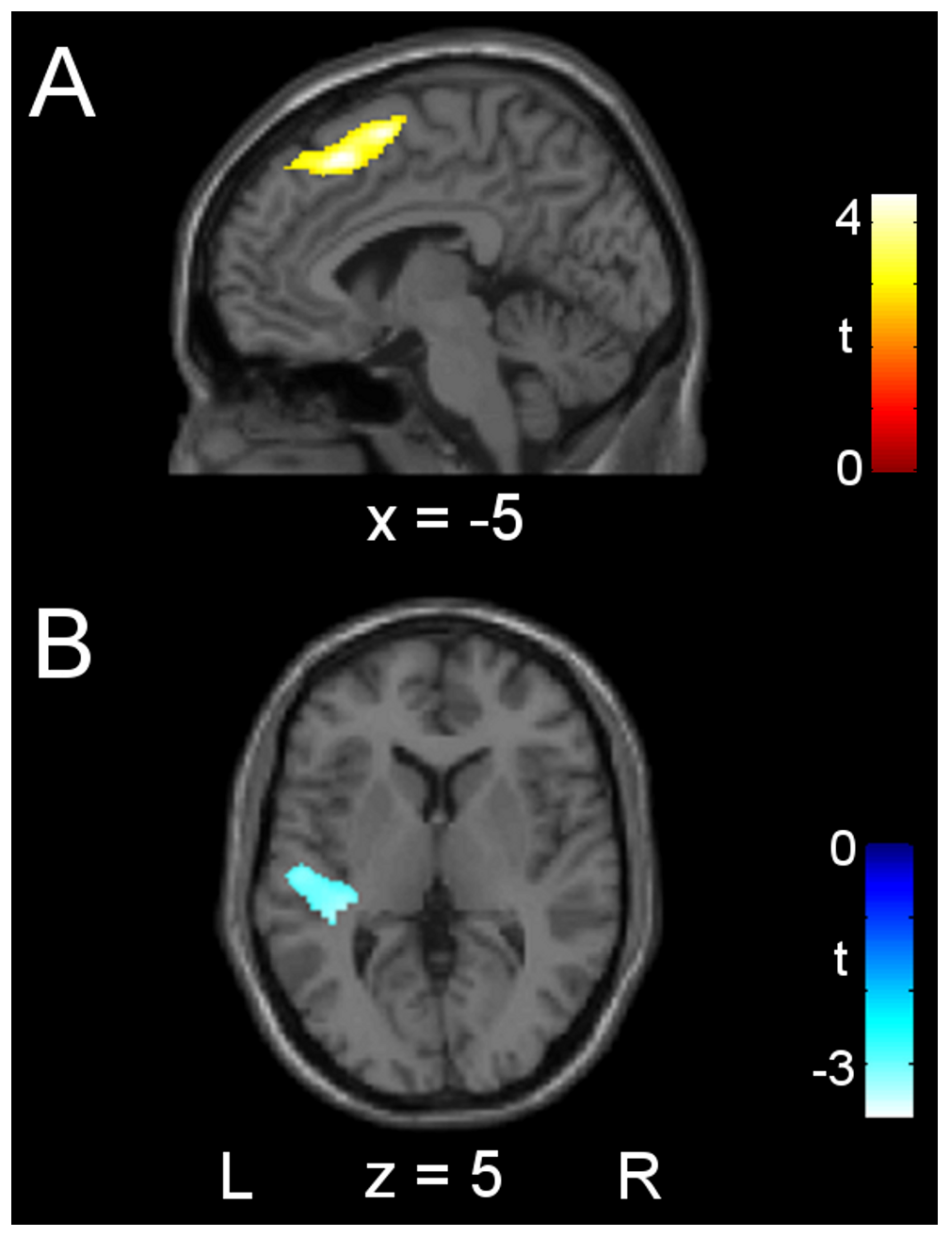
Inter-individual differences in local gray matter volume related to hypnotic suggestibility. Positive correlations of hypnotic suggestibility (HS) with gray matter volume are shown in panel A, and negative correlations in panel B. x- and z-coordinates are expressed in mm and refer to the Talairach space. L  =  left hemisphere; R  =  right hemisphere.

Two regions showed significant HS-related differences in GMV, as shown in Fig. 1. HS was positively correlated with GMV in a large cluster including the left superior and medial frontal gyri (BA 8 and 6; Talairach peak coordinates x =  −4, y =  26, z =  46; subpeak x =  −6, y =  9, z =  56), and negatively correlated with GMV in a cluster in the left superior temporal gyrus and insula (BA 41 and 13; Talairach peak coordinates x =  −32, y =  −19, z =  20).

### Functional connectivity among resting state networks

Seed-based analysis detected no significant HS-related FC for the areas identified in the VBM analysis.

**Figure 2:**
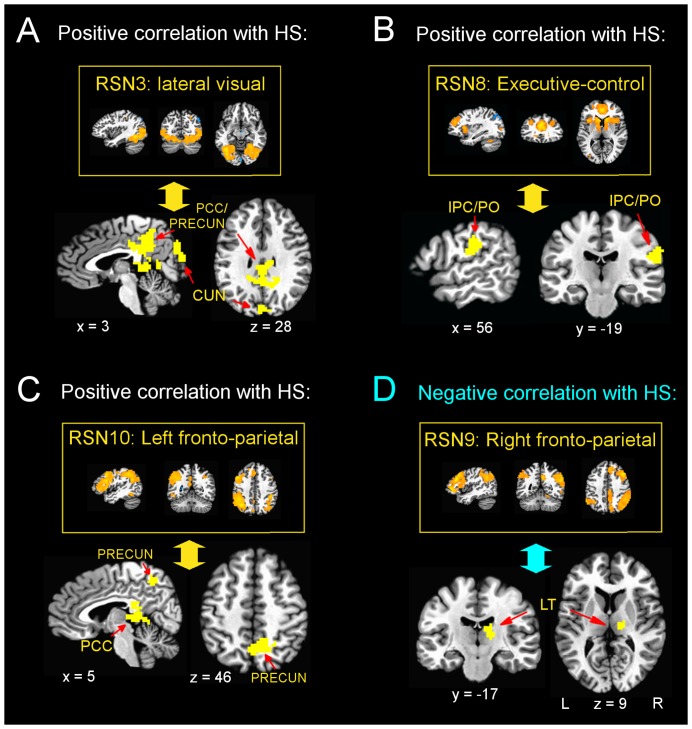
Inter-individual differences in resting state functional connectivity related to hypnotic suggestibility. Four resting state networks (RSN) showed differences in functional connectivity related to hypnotic suggestibility (HS): RSN3: lateral visual network (panel A); RSN8: executive-control network (panel B); RSN10: left fronto-parietal network (panel C). RSN9: right fronto-parietal network (panel D). x-, y- and z-coordinates are expressed in mm and refer to the Talairach space. L  =  left hemisphere, R  =  right hemisphere. PCC  =  posterior cingulate cortex; PRECUN  =  precuneus; CUN  =  cuneus; IPC  =  inferior parietal cortex; PO  =  parietal operculum; LT  =  lateral thalamus.

Four out of the ten analysed RSNs showed significant HS-related differences in FC, as shown in Table 1 and Fig. 2. Individuals with higher HS showed higher FC of the lateral visual cortex RSN (RSN3 in [17]) with clusters located in the bilateral posterior cingulate (BA 31) and cuneus/precuneus (BA 19, 7, 18; Fig. 2A), and of the executive-control RSN (RSN8 in [17]) with a right postcentral/inferior parietal cluster (BA 40, 2; Fig. 2B). HS was also positively correlated with FC between the *left* fronto-parietal RSN (RSN10 in [17]) and clusters in the bilateral posterior cingulate cortex (BA 23) and precuneus (BA 7) – Fig. 2C. By contrast, HS was *negatively* correlated with FC between the *right* fronto-parietal network (RSN9 in [17]) and the right lateral thalamus and caudate (Fig. 2D).

**Table 1:** RSNs showing significant HS-related differences in functional connectivity (results averaged across the two functional runs).

RSN	HS-contrast	Regions	BA	N° voxels	Talairach coordinates (peak)		
					x	y	z
*RSN3 (lateral visual network)*:							
	HS>0	R/L Posterior Cingulate, R/L Precuneus	31	468	−2	−35	6
	HS>0	L/R Cuneus, L Precuneus	19, 7, 18	184	−8	−89	12
*RSN8 (executive control network)*							
	HS>0	R Inferior Parietal Lobule, R Postcentral Gyrus	40, 2	115	56	−20	24
*RSN9 (right fronto-parietal network)*:							
	HS<0	R Thalamus, R Caudate		103	11	−20	18
*RSN10 (left fronto-parietal network)*:							
	HS>0	R/L Posterior Cingulate	23	144	5	−32	21
	HS>0	R/L Precuneus	7	93	11	−44	39

HS > 0, positive correlation between hypnotic suggestibility (HS) and functional connectivity (FC); HS < 0, negative correlation between HS and FC; RSN, Resting State Network; BA, Brodmann Area, R, right; L, left.

## Discussion

Our results show that, in healthy women, HS is associated with inter-individual differences in cerebral structure as well as in functional connectivity at rest of several brain areas involved in self-processing, awareness, attentional control and imagery.

### Differences in gray matter volume related to hypnotic suggestibility

To our knowledge, this is the first study demonstrating HS-related differences in local gray matter volume. A positive correlation was found in the left superior and medial frontal gyri (BA 8, 6), including the supplementary motor area (SMA) and pre-SMA. The greater GMV may reflect a neurotrophic/plasticity effect due to higher/more frequent activity in these areas in individuals with higher HS [32]. SMA is involved in the control of movement, including the postural stabilization of the body; pre-SMA is associated with cognitive aspects of a variety of tasks, such as establishing or retrieving sensory-motor associations and processing or maintenance of relevant sensory information [33]. Interestingly, postural control and locomotion, sensory-motor integration and cross-modal object recognition are more effective/flexible in Highs compared to Lows [6], [34], [35].

HS was also *negatively* correlated with GMV in a cluster including the left posterior insula and superior temporal gyrus (STG). The insula integrates external sensory input with the limbic system and is integral to the awareness of the body's state (interoception) and the sense of self [36], [37]. The posterior insula is specialised for multimodal sensory processing, and specifically for the sensory-discriminative aspects of pain [38]. Structural neuroimaging studies have consistently found decreased gray matter in the bilateral STG and insula in patients with schizophrenia; these alterations may be related to symptoms such as difficulty in distinguishing between self-generated and external sensory input, leading to hallucinations [37], [39]. Interestingly, HS is associated with the personality trait of schizotypy and with an increased risk of developing schizophrenia, which shares several characteristics with the hypnotic state, including hallucinations/hallucination-like experiences and a reduced sense of agency [40].

Together with previous findings showing a greater size of the anterior corpus callosum in Highs compared to Lows [41], our results suggest that some specific cognitive correlates of HS are reflected by, and possibly based upon, structural variability in discrete brain regions.

The observed structural differences in GMV were not accompanied by any HS-related differences in resting state functional connectivity (FC) of the same regions.

### Differences in functional connectivity related to hypnotic suggestibility

In our study, we aimed to relate HS to FC of different RSNs, which have been found to exhibit spatial correspondence with broadly defined functional circuits [17].

Individuals with higher HS showed higher FC between medial posterior areas involved in vision and imagery [42] – including bilateral posterior cingulate cortex (PCC), precuneus and cuneus – and both the lateral visual network (RSN3, which includes non-primary visual areas) and the left fronto-parietal network (RSN10); this network includes the DLPFC and is involved in cognitive control processes, such as integrating information from the external environment with stored internal representations to guide decisions and performance adjustments [43], [44]. The left fronto-parietal network also includes Broca's and Wernicke's areas, classically implicated in language processing [17].

In contrast, subjects with higher HS showed *lower* FC between the *right* fronto-parietal network (RSN9), which has been associated with somesthesis and pain [17], and the right lateral thalamus/caudate, receiving peripheral somatosensory input. Interestingly, the right fronto-parietal network also partly overlaps with a right hemisphere-dominant ventral fronto-parietal attention network, which is responsible for reorienting attention towards unexpected but important environmental stimuli, and which is suppressed when attention is focussed to prevent reorienting to distracting events [45].

Finally, HS was positively correlated with FC between the executive-control network (RSN8), which includes anterior cingulate cortex (ACC), paracingulate cortex and anterior insula and is involved in cognition, emotion and perception/somesthesis/pain [17], and a right postcentral/inferior parietal cluster (BA 40, 2), namely a region involved in somatosensory processing.

Overall, the present findings are consistent with a cognitive scenario of greater engagement at rest of imagery and self-monitoring processes in women with higher HS scores, with a reduced contribution of sensory thalamic input, which may reflect a higher absorption in mental activity and a lower distractibility by external stimuli. This hypothesis is in line with research showing that high HS is associated with highly vivid imagery [18] and fantasy-proneness [19], and with some measures of mental absorption [1], [23].

Hoeft et al. [13] recently explored differences between Highs and Lows in waking resting state FC of a “salience network” including the dACC, frontoinsular cortices and limbic structures, the default mode network, and left and right fronto-parietal networks (included in a single template in that study). They found higher FC in Highs compared to Lows between the left DLPFC and the salience network, and in particular between the left DLPFC and the dorsal ACC.

We extend their findings by demonstrating HS-related differences in FC in posterior brain regions involved in vision and imagery, confirming our second hypothesis. However, contrary to our first hypothesis, we could not replicate Hoeft et al.'s finding of HS-related differences in FC between left DLPFC and ACC. These differences could be ascribed to various reasons, e.g.: the fact that we used different RSN templates; that we studied only women; and that our sample includes subjects with medium HS scores, in addition to Highs and Lows.

Further research is needed to explore the relationship between HS-related differences observed in waking, and the neural bases of the phenomena observed in the hypnotic state. Recent neuroimaging studies have emphasized the role of the DLPFC and ACC/mid-cingulate cortex (MCC) in hypnotic response [1], [14]. Interestingly, the precuneus may also play a role in hypnotic responses. This region is involved in visuo-spatial imagery, episodic memory retrieval, self-processing and consciousness [46] and shows reduced activity during altered states of consciousness, including the hypnotic state [47]. In healthy Highs, hypnotic paralysis of the left hand was shown to be associated with increased FC of the precuneus with the right DLPFC and angular gyrus [48]. We recently reported HS-related differences in neural activity mediating the placebo analgesic response in several areas, including the precuneus [49]. These findings suggest that the precuneus may play a role in maintaining a modified representation of the self in response to suggestions.

### Imitations

This study has some limitations. First, we chose to study a random sample, in order to reflect the distribution of HS in the general population; however, as this includes only 10–15% Highs [50], a more specific assessment of the effects reported in the present paper in the higher portion of the HS range will necessarily have to employ a targeted pre-selection of the volunteers or a much larger general-population sampling.

Second, our sample included only women. Future studies including both genders are needed to extend our conclusions to the general population.

Third, we assessed only *linear* relationships with HS, in line with previous behavioural studies, which have documented linear associations between HS and other cognitive constructs, such as fantasy proneness and mental absorption [1]. However, as HS appears to be a multidimensional trait [51], its links to brain function and structure are likely complex as well, and future research will be able to better assess the non-linear and multivariate nature of the relationship.

## Conclusion

Our results demonstrate that the cognitive trait of HS is associated both with structural differences in GMV in cortical areas related to motor control, sensory-motor integration and interoception, and with differences in resting state FC in frontal attentional networks and in medial posterior areas involved in imagery. Additional research is needed to confirm these relationships also in males, and to investigate HS-related differences in FC of specific task-related cortical networks [52], as well as their cognitive correlates.
